# Prediction of Surgical Approach in Mitral Valve Disease by XGBoost Algorithm Based on Echocardiographic Features

**DOI:** 10.3390/jcm12031193

**Published:** 2023-02-02

**Authors:** Xiaoxuan Lin, Lixin Chen, Defu Zhang, Shuyu Luo, Yuanyuan Sheng, Xiaohua Liu, Qian Liu, Jian Li, Bobo Shi, Guijuan Peng, Xiaofang Zhong, Yuxiang Huang, Dagang Li, Gengliang Qin, Zhiqiang Yin, Jinfeng Xu, Chunying Meng, Yingying Liu

**Affiliations:** 1 Department of Ultrasound, The Second Clinical Medical College, Jinan University (Shenzhen People’s Hospital), Shenzhen 518020, China; 2 Department of Cardiovascular Surgery, Shenzhen People’s Hospital (The Second Clinical Medical College, Jinan University; The First Affiliated Hospital, Southern University of Science and Technology), Shenzhen 518020, China

**Keywords:** mitral valve disease, echocardiography, prediction, XGBoost, mitral valve repair, mitral valve replacement

## Abstract

In this study, we aimed to develop a prediction model to assist surgeons in choosing an appropriate surgical approach for mitral valve disease patients. We retrospectively analyzed a total of 143 patients who underwent surgery for mitral valve disease. The XGBoost algorithm was used to establish a predictive model to decide a surgical approach (mitral valve repair or replacement) based on the echocardiographic features of the mitral valve apparatus, such as leaflets, the annulus, and sub-valvular structures. The results showed that the accuracy of the predictive model was 81.09% in predicting the appropriate surgical approach based on the patient’s preoperative echocardiography. The result of the predictive model was superior to the traditional complexity score (81.09% vs. 75%). Additionally, the predictive model showed that the three main factors affecting the choice of surgical approach were leaflet restriction, calcification of the leaflet, and perforation or cleft of the leaflet. We developed a novel predictive model using the XGBoost algorithm based on echocardiographic features to assist surgeons in choosing an appropriate surgical approach for patients with mitral valve disease.

## 1. Introduction

Cardiac valves are the central components responsible for the efficient functioning of circulation. Owing to longer life spans and the aging population, the prevalence of heart valve disease is increasing worldwide, with mitral valve regurgitation being one of the most common heart valve diseases [1,2]. A global epidemiological analysis of heart valve diseases published in 2021 suggested that approximately 24.2 million people worldwide suffer from mitral valve regurgitation, and it is estimated that this disease directly caused more than 30,000 deaths in 2019 [3]. Mitral valve regurgitation is divided into primary and secondary mitral valve regurgitation based on etiology. Primary mitral valve regurgitation is due to structural abnormalities of the mitral valve apparatus, whereas secondary mitral valve regurgitation is due to structural changes in the left ventricle and atrium. In Western countries, primary mitral valve regurgitation is commonly caused by degenerative changes (such as fibroelastic deficiency and Barlow disease), but rheumatic heart valve disease is the most common cause of mitral valve regurgitation in developing countries. Infective endocarditis can also lead to mitral valve regurgitation [1,3,4].

Patients who suffer from severe mitral valve regurgitation will require surgical treatment in the form of mitral valve replacement or repair. According to the Guidelines for the Management of Valvular Heart Disease [4,5], mitral valve repair is the first choice for surgical intervention in patients who are being considered for surgery. Mitral valve repair of the patient’s native valve and sub-valvular structures better preserves the patient’s left ventricular function [4,5,6] and avoids or reduces many of the complications associated with prosthetic valves, such as endocarditis, prosthetic valve dysfunction, thromboembolism, or pacemaker implantation [7], thereby greatly improving the patient’s postoperative quality of life. Patients who underwent mitral valve repair had better mortality rates than those who underwent mitral valve replacement, as assessed by an analysis of the relevant cardiac surgery literature [7,8,9].

Nevertheless, mitral valve repair is not appropriate for all patients with mitral valve disease and the procedure will increase the risk of secondary cardiopulmonary bypass and postoperative recurrence if performed in an unsuitable patient, which can have a detrimental effect on the patient. Echocardiography can determine the etiology and involvement of the lesion, leaflets, and accessory structures. According to ASE guidelines [4,10], echocardiography, particularly transesophageal echocardiography (TEE), is the best evaluation method for valvular heart disease. At the same time, a comprehensive evaluation of the size of the heart chambers and cardiac function can be provided to surgeons as a comprehensive basis for surgical planning. The guidelines indicate that preoperative evaluation by echocardiography is essential in choosing the mitral valve surgical approach [4]. However, the indicators of echocardiographic features have an impact on the choice of procedure and are not uniformly defined across studies [7,11,12,13].

In recent years, a novel tree structure algorithm, XGBoost, has been widely used in classification and regression tasks [14]. Its advantages include, firstly, good performance and robustness [15], as verified by a large number of applications in data science (such as Kaggle). A second advantage is convenient transplantation. XGBoost can be run on various distributed environments, such as Kubernetes, Hadoop, SGE, etc. Additionally, the importance of influencing factors is provided [16]. This information can provide auxiliary references for data understanding and modification of task schemes.

Therefore, our study attempted to create a predictive model using the XGBoost algorithm based on the assessment of the mitral valve apparatus in preoperative echocardiography, such as the annulus, leaflets, and sub-valvular structures. Consequently, the model provides a simplified, feasible, and highly efficient assessment of the surgical options (repair or replacement) for patients with mitral valve disease.

## 2. Methods

### 2.1. Study Design

**Figure 1:**
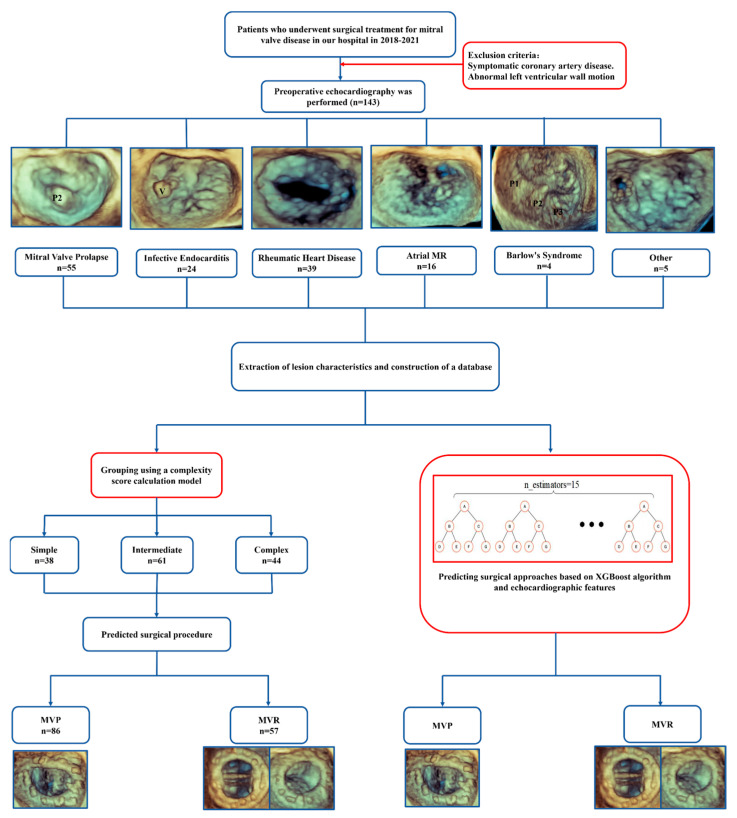
Flow chart of patient selection to establish a predictive model. Abbreviations: Atrial MR: atrial mitral regurgitation; MVP, mitral valve repair; MVR, mitral valve replacement.

We retrospectively analyzed consecutive patients who underwent mitral valve surgery for mitral valve disease between 2019 and 2021 (Figure 1). A total of 143 patients were included; 86 underwent mitral valve repair and 57 underwent mitral valve replacement. Each patient was assigned a complexity score based on weighted intraoperative anatomical characteristics and a technique score indicating the number of techniques used to complete the surgery. Patients were categorized into 3 groups based on their complexity score and comparisons were made among the groups for technique score and surgery treatment. We used the XGBoost algorithm to establish a prediction model for the selection of lesions to guide the individualization of clinical treatment options.

The inclusion criteria for this study were patients with mitral valve disease requiring surgical intervention, including mitral valve prolapse, Barlow’s syndrome, rheumatic heart valve disease, infective endocarditis, atrial mitral regurgitation, and other causes of mitral valve regurgitation or stenosis. The exclusion criteria were previous mitral valve surgery or interventional therapy and secondary mitral valve regurgitation in the absence of mitral valve anatomical changes.

Patients with concomitant tricuspid annuloplasty, surgical ablation for atrial fibrillation, and coronary artery disease, which was found incidentally during preoperative catheterization, were included in the study. However, patients presenting with symptomatic coronary or left ventricular wall motion abnormalities were excluded.

### 2.2. Complexity and Technique Scores

We assessed the presence or absence of mitral valve disease and degree of dysfunction using the patient’s preoperative echocardiography. The leaflets, annulus, and subvalvular structures of the mitral valve were evaluated. The following lesions or dysfunctions were chosen for inclusion in the complexity score: presence or absence of prolapse in the 8 leaflet segments; restriction or hypermobility of any leaflet segment; calcification of the annulus; calcification of leaflets or subvalvular structure; commissure fusion; and presence of ruptured chordae tendineae. We expected these lesions and dysfunctions to be routinely investigated using good-quality echocardiography.

**Figure 2:**
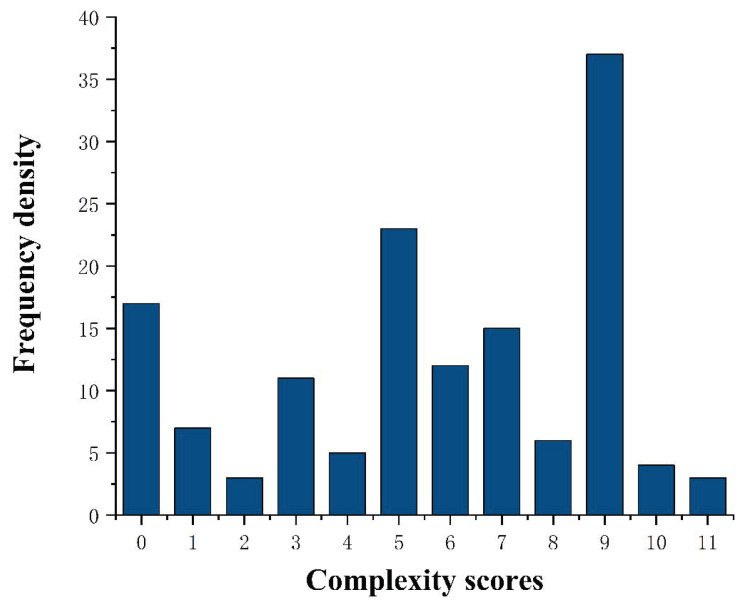
Frequency chart of complexity scores for enrolled patients.

We arbitrarily assigned a weight to each of these variables (Table 1). Weights were assigned as follows: posterior leaflet prolapse (score of 1 for each prolapsing segment [P1, P2, and P3]; score of 0 for nonprolapsing segments; multisegment prolapse was scored as the sum of the scores for the prolapsing segments); anterior leaflet prolapse (score of 2 for each prolapsing segment [A1, A2, and A3]; score of 0 for nonprolapsing segments; multisegment prolapse was scored as the sum of the scores for the prolapsing segments); commissural prolapse (score of 2 for each prolapsing commissure [anterolateral and posteromedial]; score of 0 for nonprolapsing commissures); ruptured chordae tendineae (score of 1 for the presence of ruptured chordae tendineae); leaflet morphology (score of 1 for leaflet thickening; score of 3 for redundant leaflet); calcification (score of 1 for calcification of the leaflet; score of 3 for calcification of the annulus or chordae tendineae; score of 0 for no calcification); commissure fusion (score of 2 if present); vegetation, perforation, or cleft (score of 1 if only one was present; score of 4 if more than one was present); and leaflet motion (score of 1 if hypermobility was present in any valve segment; score of 2 if restriction was present in any valve segment; score of 0 if neither were present in any valve segment; multisegment restriction or hypermobility was assigned a single score of 2). Complexity scores were entered into a frequency chart (Figure 2). Based on an examination of the complexity score distribution, we defined 3 complexity groups: simple (complexity score 1–4), intermediate (complexity score 5–8), and complex (complexity score ≥ 9).

The technique score was calculated by adding the number of principal techniques used to repair each valve. The techniques included in the calculation were as follows: annuloplasty, chordal replacement with either artificial or native chords (multiplied by the number of segments resuspended by chords), leaflet cleft closure, leaflet resection, vegetation removal, and commissurotomy. Valve re-repair was counted as a separate technique in addition to any other techniques employed. When revision of the annuloplasty ring was required during re-repair, an additional score of 1 was added. The sum of each surgical technique was the surgical technique score for the patient [17]. For example, the surgical technique score would be 3 (1 × 2 + 1) if a patient underwent mitral valve repair for mitral prolapse and two artificial chordae tendineae and an annuloplasty ring were implanted during the operation.

### 2.3. Endpoint

Successful repair was defined as less than mild mitral regurgitation on the postoperative echocardiogram based on the grade defined by the guidelines of the American Society of Echocardiography [4,5]. Adverse events were defined as death during postoperative hospitalization, unsatisfactory intraoperative plasticity, conversion to replacement surgery (intraoperative water pumping test in patients with large regurgitation or intraoperative transesophageal echocardiography in patients with mild or moderate regurgitation), and mild symptoms during follow-up.

### 2.4. Database Setup

The database was established based on the above data, and the number of classifications is shown in Table 2. The detailed parameters of the original sample population are shown in Table 3. Through empirical analysis, these variables were divided into effective, ineffective, and uncertain variables according to their impact on the operation method. A previous study indicated that repair of the anterior leaflet is more difficult than that of the posterior leaflet, but there was a lack of evaluation of the impact of each segment. The impact of each segment on mitral valve repair is unclear. Therefore, we defined prolapsed segments as an uncertain variable. Invalid variables were removed, reducing the debugging complexity.

### 2.5. Statistical Analysis

Continuous variables were reported as the mean with standard deviation or median with interquartile range. Categorical variables were expressed as a proportion. Using the lesion score calculated by the established lesion scoring system and SPSS statistical software (SPSS, version 26.0, Inc, Chicago, IL, USA), the ROC curve was drawn and the best cutoff value was selected.

The distributions of the echocardiographic features of mitral valve disease were entered, a database was established, and XGBoost was used to build a prediction model. All experimental codes were implemented based on Python language under the sklearn library. The model setting adopted the K-fold crossover method (K = 3) and the data for this study were obtained based on triplicate experiments. The dataset was randomly divided into three subsets on average: groups 0, 1, and 2. Each subset was used as the test set and the remaining two subsets were used as the training set. Training was carried out three times and indicators for evaluating the model were obtained. The hyperparameter settings of the model were as follows: the number of base classifiers was set to 15, the learning rate was set to 0.3, and the maximum tree depth was set to 2. The model was evaluated for accuracy (ACC), which was defined as the ratio of the number of correctly predicted samples to the total number of samples. ACC was calculated as follows:

$$\mathrm{Acc}=\frac{\mathrm{TP}+\mathrm{TN}}{\mathrm{TP}+\mathrm{FN}+\mathrm{FP}+\mathrm{TN}}$$

where TP (true positive) is the number of samples that are correctly classified as positive; FN (false negative) is the number of samples that are incorrectly predicted to be negative; FP (false positive) is the number of samples that are incorrectly predicted to be positive, and TN (true negative) is the number of samples that are correctly classified as negative. To fully display the prediction results of the model, this paper included the confusion matrix of the prediction results to accurately quantify the true-positive, true-negative, false-positive, and false-negative test samples.

Due to the scarcity of medical data, relatively few databases have been constructed. Therefore, our study adopted the K-fold crossover method (K = 3) to divide the data into 3 groups of roughly equal subdata, and the sample sizes were 48, 48, and 47 for Groups 0, 1, and 2, respectively (the corresponding sequences were generated by pseudo random numbers, and the initial seed was set to 0 to achieve reproducibility of sample grouping). Each of the groups was set as the test group in turn, and the remaining two groups were set as the training group, for a total of 3 experiments. The data of the unspecified groups in this paper were based on the average of the results of three experiments.

## 3. Results

### 3.1. Complexity Score and Patient Characteristics

**Figure 3:**
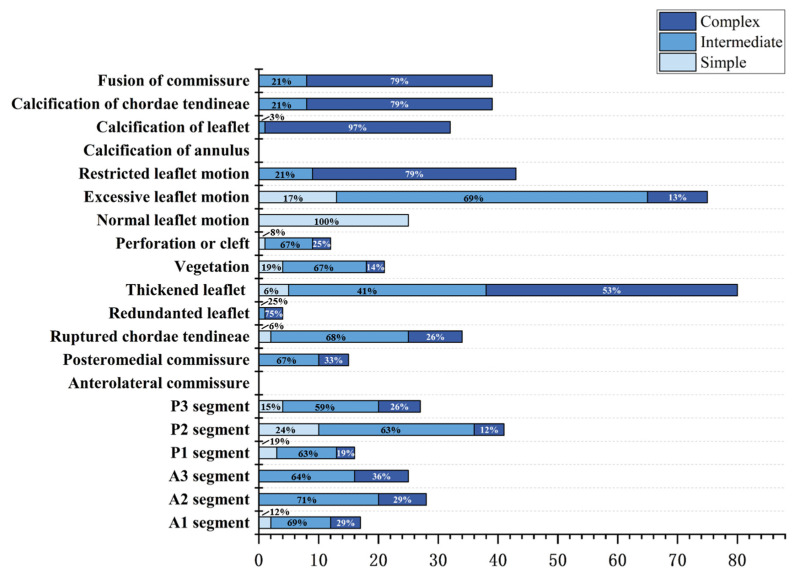
Histogram of echocardiographic features in three different groups.

**Figure 4:**
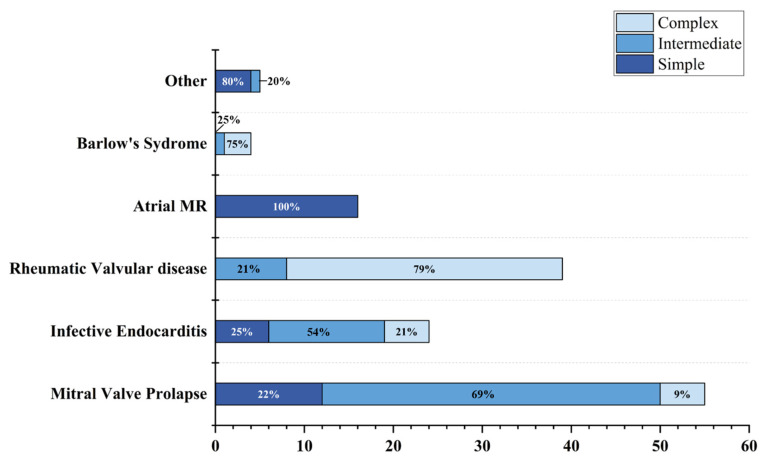
Histogram displaying the distribution of etiologies in three different groups. Abbreviations: Atrial MR: Atrial mitral regurgitation.

A total of 143 patients were included in this study, and the frequency chart of the complexity scores is shown in Figure 2. The detailed frequency of individual echocardiographic features is shown in Table 3 and Figure 3. In the study population, 38 patients (26.6%) were in the simple group, 61 patients (42.6%) were in the intermediate group, and 44 patients (30.8%) were in the complex group. 31 patients (70.5%) in the complex group had rheumatic valve disease. Atrial mitral regurgitation due to annular enlargement was the most common etiology in the simple group and mitral valve prolapse was the most common etiology in the intermediate group. The frequency of each etiology for each group is shown in Figure 4.

Involvement of the posterior valve was most commonly observed in patients with mitral valve prolapse, with the majority in the P2 segment [41/55 (74.5%)]. This distribution is related to the anatomy of the mitral valve and is consistent with previous studies [17,18,19]. Calcification of the mitral valve and its appendages did not occur in the simple group, with leaflet thickening, subvalvular structure contracture, calcification, and commissural fusion being the most common features. Commissural fusion is characteristic of rheumatic valvular heart disease [20,21].

### 3.2. Repair Feasibility and Surgical Outcomes

**Figure 5:**
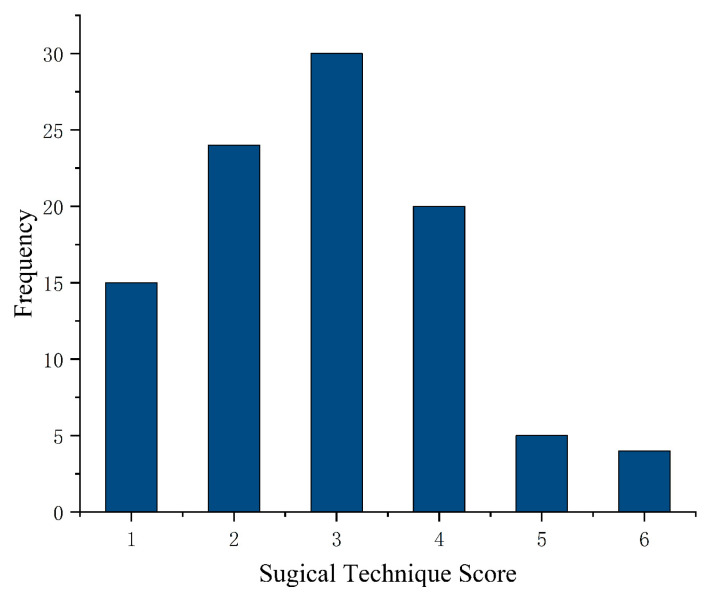
Frequency of surgical technique scores for patients who underwent mitral valve repair.

**Figure 6:**
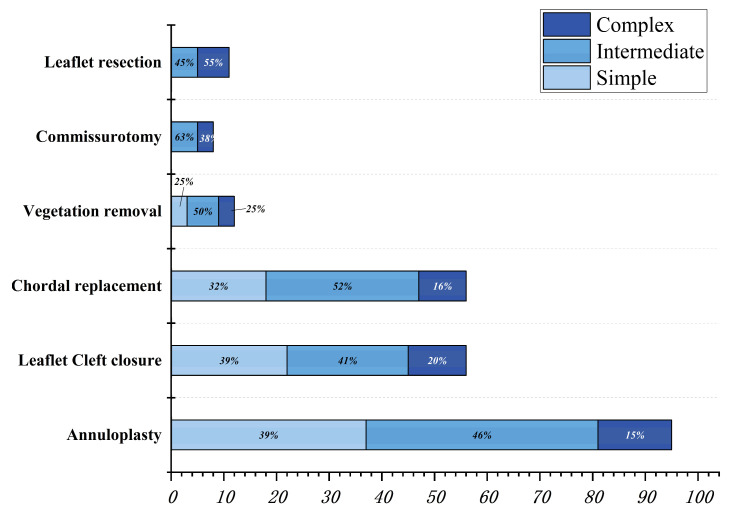
Histogram displaying the distribution of different surgical techniques for three groups of patients who underwent mitral valve repair.

All patients who underwent mitral valve repair had good surgical results, except for 15 (15%) patients. Eleven patients were diverted to mitral valve replacement due to excessive regurgitation at the time of intraoperative assessment. Four patients developed more than mild regurgitation during follow-up (Table 4). The mitral valve repair technique score was positively correlated with the complexity of the lesion (r = 0.6317, *p* < 0.0001) (Supplementary Materials Figure S1). Frequency charts of the surgical technique scores of patients who underwent mitral valve repair and the use of each specific technique are shown in Figure 5 and Figure 6.

Almost all patients who underwent mitral valve repair had an annuloplasty ring implanted to reduce the area of the annulus and improve coaptation, thus reducing or even eliminating regurgitation. In rheumatic heart valve disease, commissure fusion occurred, and the mitral valve commissure was incised to expand the valve area and reduce the degree of valve stenosis.

### 3.3. Case Example

The following are the specific circumstances of four cases included in this analysis.

**Figure 7:**
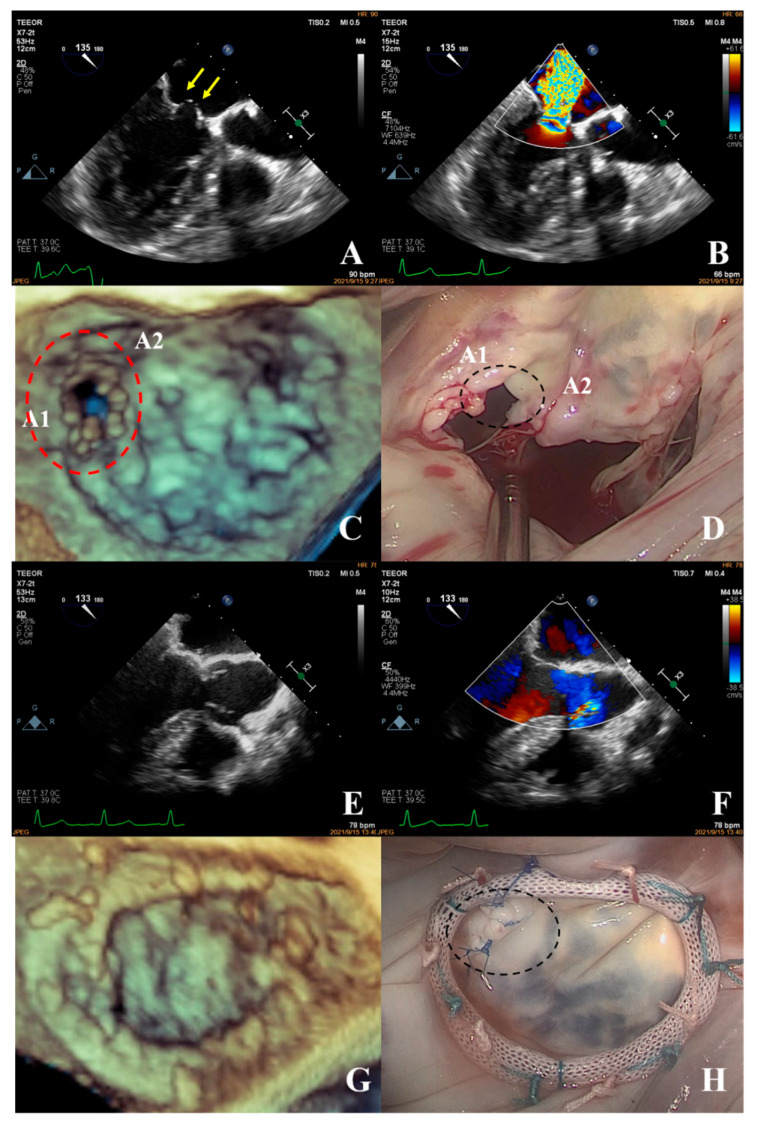
Prolapse of the anterior mitral valve with apparent discontinuity (**A**) (yellow arrow shows the cleft of the leaflet), massive regurgitation during systole, VC: 8.0 mm (**B**); the three-dimensional image of the mitral valve shows a cleft (**C**) between the A1 and A2 segments (dotted red line). During surgery, a cleft (**D**) was observed between the A1 and A2 segments of the anterior mitral leaflet, consistent with the preoperative diagnosis. The cleft was sutured and the mitral valvular ring (**H**) was placed. The surgical technique score was 1 + 1 = 2. After the spontaneously returned heartbeat, the forming effect was good and no obvious reflux signal (**E**,**F**) was found. The annuloplasty ring of the mitral valve was visible on 3D ultrasound (**G**), which was consistent with the intraoperative view (**H**).

**Figure 8:**
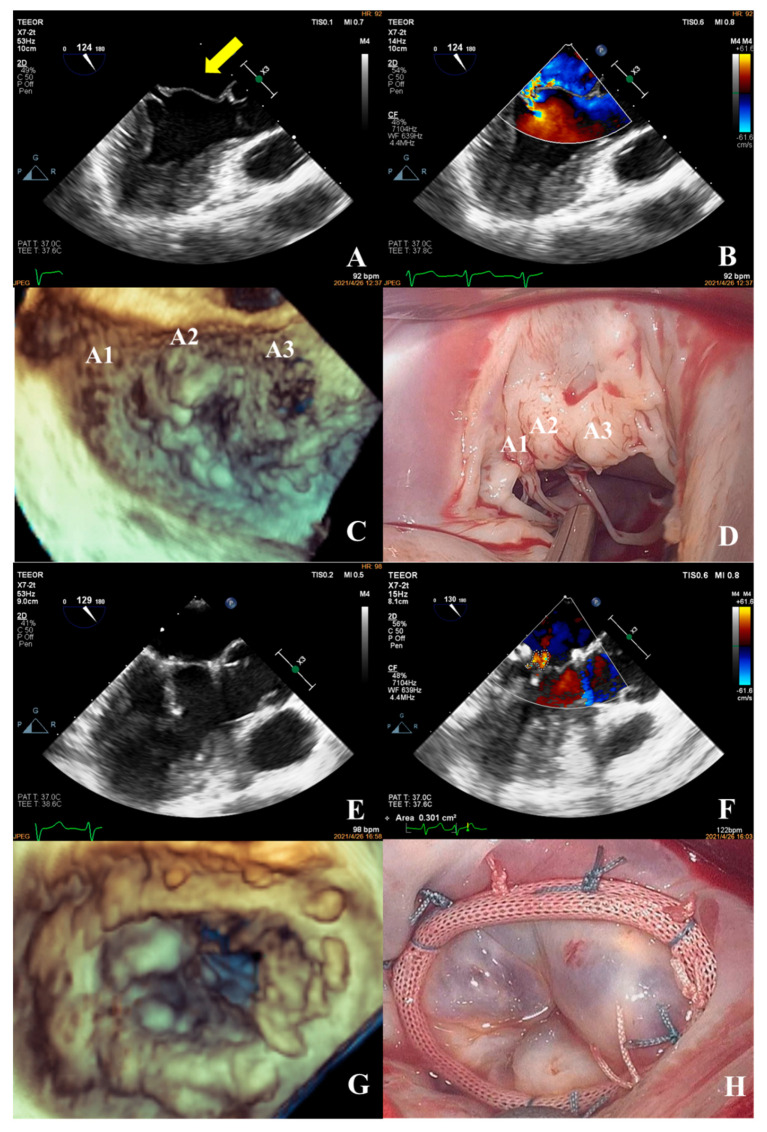
Transesophageal echocardiography was performed; the anterior mitral valve leaflet prolapsed into the left atrium in diastole (**A**) (yellow arrow shows prolapse of the anterior leaflet) and a severe eccentric regurgitant signal was seen in systole (VC: 5.5 mm) (**B**). Prolapse of the mitral valve in the anterior leaflet A1, A2, and A3 segments was observed in 3D images (**C**). Intraoperative confirmation of prolapse in the A1, A2, and A3 segments of the anterior mitral valve leaflet (**D**) was consistent with the preoperative diagnosis. Intraoperatively, two artificial chordae were reconstructed and a mitral valvuloplasty ring was implanted. After resuscitation, the result of the repair was good, with a height of coaptation of 6.4 mm (**E**), and a tiny regurgitant signal was observed in diastole, with a regurgitant area of 0.3 cm^2^ (**F**). The annuloplasty ring of the valve was visible on 3D ultrasound (**G**), which was consistent with the intraoperative view (**H**).

**Figure 9:**
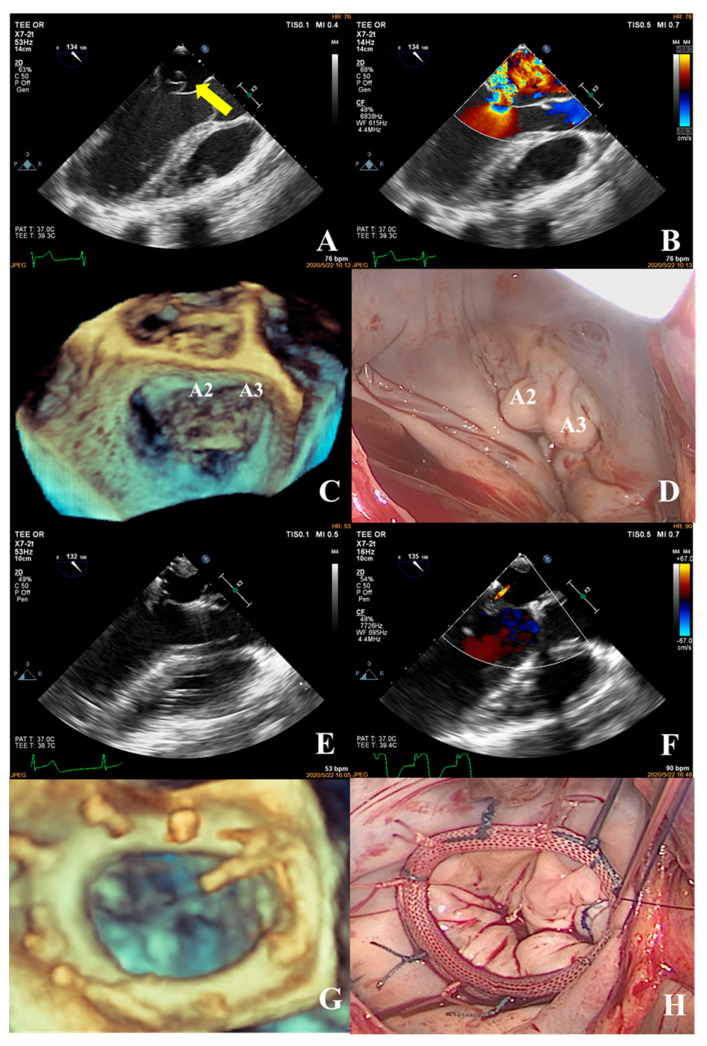
Transesophageal echocardiography was performed; the anterior mitral leaflet prolapsed into the left atrium in diastole (**A**) (yellow arrow shows the segment of anterior leaflet prolapse) with a redundant leaflet and a large regurgitant signal in systole (VC: 8.6 mm) (**B**). Mitral valve prolapses in the A2 and A3 segments of the anterior leaflet involving the posteromedial commissure were observed in 3D images (**C**). Intraoperative confirmation of prolapse in the A2 and A3 segments of the anterior mitral leaflet (**D**). This was consistent with the preoperative diagnosis. Intraoperatively, two artificial tendon cords were placed in the A2 and A3 segments and a mitral annuloplasty ring was implanted. After resuscitation, a good surgical result was observed, the height of the coaptation edge was 6.4 mm (**E**), and a tiny regurgitant signal was seen in diastole (**F**), with a regurgitant area of 0.8 cm^2^. The annuloplasty ring of the mitral valve was visible on 3D ultrasound (**G**), which was consistent with the intraoperative view (**H**).

**Figure 10:**
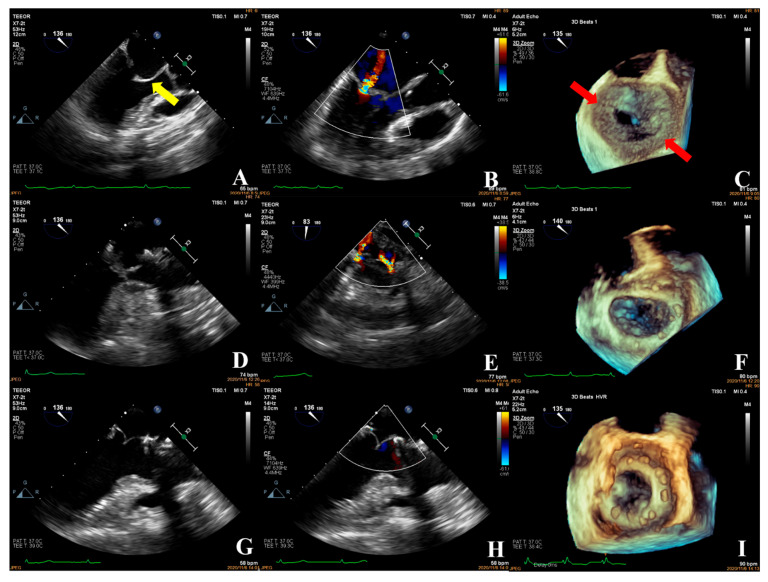
The patient had rheumatic valvular heart disease with thickened leaflets and restricted movement (**A**) (yellow arrows show thickened leaflets), thickening and shortening of the chorda tendineae, and moderate systolic regurgitation (VC: 3.8 mm) (**B**). A three-dimensional image of the mitral valve showed commissure fusions (**C**) (red arrow shows commissure fusion). The complexity score was 1 + 3 + 2 + 2 = 8, which was considered as the intermediate group. The commissure of both sides was cut open during the operation and the annular ring was placed. The surgical technique score was 2 × 2 + 1 = 5. The intraoperative TEE examination (**D**,**F**) showed two regurgitant jets (**E**), starting from the anterolateral and posterolateral commissure, respectively, with a regurgitant area of approximately 2.55 cm^2^. The forming effect was poor and required a second bypass run. After the heartbeat spontaneously returned, intraoperative TEE examination showed good function of the prosthetic valve (**G**–**I**).

- In the simple group, the etiology was primarily secondary annular enlargement or mitral valve prolapse in an isolated segment leading to mitral regurgitation. The most commonly used technique was annuloplasty. The coaptation area of the valve leaflets was increased to reduce or even eliminate mitral regurgitation. For example, the complexity score was 3 (2 + 1) for a patient with A1 segment prolapses, a mitral anterior leaflet cleft, no chordae tendineae rupture, and no leaflet calcification, which classified the patient in the simple group. The cleft was sutured during the operation and a mitral annuloplasty ring was placed. The surgical technique score was 2 (1 + 1). After resuscitation, the surgical effect was good and no obvious regurgitation signal was observed (Figure 7).
- The most common pathogenesis in the intermediate group was mitral valve prolapse involving multiple segments, with or without ruptured chordae tendineae. Mitral valve prolapses most often involved the posterior leaflet, specifically the P2 segment [17,19,22]. A patient with mitral valve prolapses involving the A1, A2, and A3 segments but no rupture of chordae tendineae had a complexity score of 7 (3 × 2 + 1) and a surgical technique score of 5 (2 × 1 + 1). The prolapsed leaflets were processed to reconstruct the artificial chordae tendineae and suture the sector junction to provide enough support when the leaflets were closed, thus increasing the coaptation area to reduce regurgitation (Figure 8).
- Patients in the complex group had a variety of etiologies, including multiple segmental mitral valve prolapse (12.5%), Barlow’s syndrome (7.1%), infective endocarditis (12.5%), and rheumatic valvular heart disease (67.9%). The proportion of patients with each of the three less common etiologies was essentially the same. These diseases not only involve a wide range of lesions but are also accompanied by changes in valve morphology and structure, which increases the difficulty of surgery. Preoperative evaluation of the lesion is also a challenge for echocardiologists. A patient with preoperative suspicion of Barlow’s syndrome exhibited prolapse involving the A2 and A3 segments and posteromedial commissure, accompanied by redundant valve leaflets. The complexity score was 9 (2 × 2 + 2 + 3). Two artificial chordae tendineae were implanted in each of the A2 and A3 segments and a mitral annuloplasty ring was placed. The surgical technique score was 5 (4 × 1 + 1) and the surgical effect was good (Figure 9).
- The fourth case was an unsuccessful mitral valve repair that was converted to mitral valve replacement (Figure 10). The patient had rheumatic valvular disease, with thickened leaflets, restricted leaflet mobility, thickened and shortened sub-valvular chordae tendineae, and commissural fusion observed on three-dimensional images. The complexity score was 8 (1 + 3 + 2 + 2). According to the surgeon’s experience, mitral valve repair was expected to be performed, but the surgical effect was not satisfactory and the patient required a second bypass run. After resuscitation, the intraoperative TEE examination showed that the function of the artificial valve was good.

### 3.4. Prediction of a Surgical Approach Based on Complexity Score

**Figure 11:**
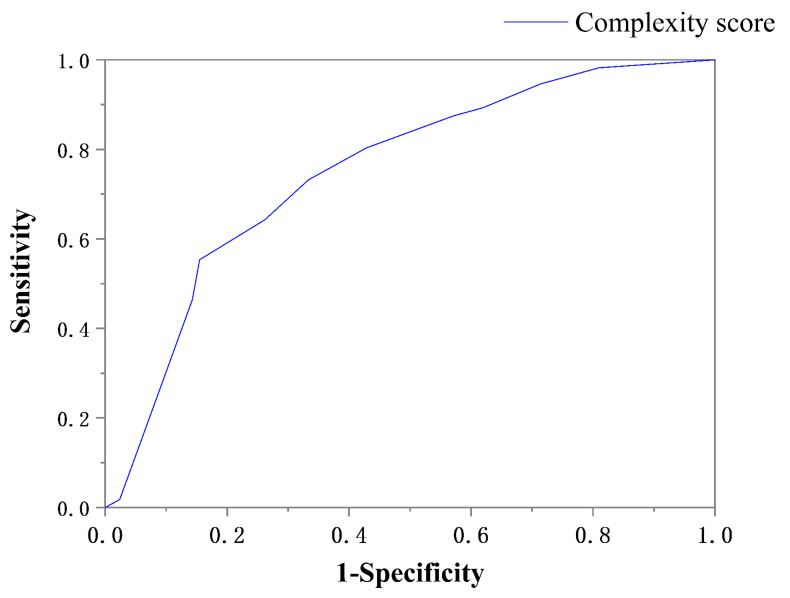
ROC curve analysis: complexity score and surgical approach.

We used the complexity score to generate an ROC curve in order to predict the appropriate surgical procedure (Figure 11). The AUC was 0.75 (95% confidence interval: 0.67–0.83) and the cutoff value was 8.5 (Table 5).

### 3.5. XGBoost Model Accuracy and Confusion Matrix

**Figure 12:**
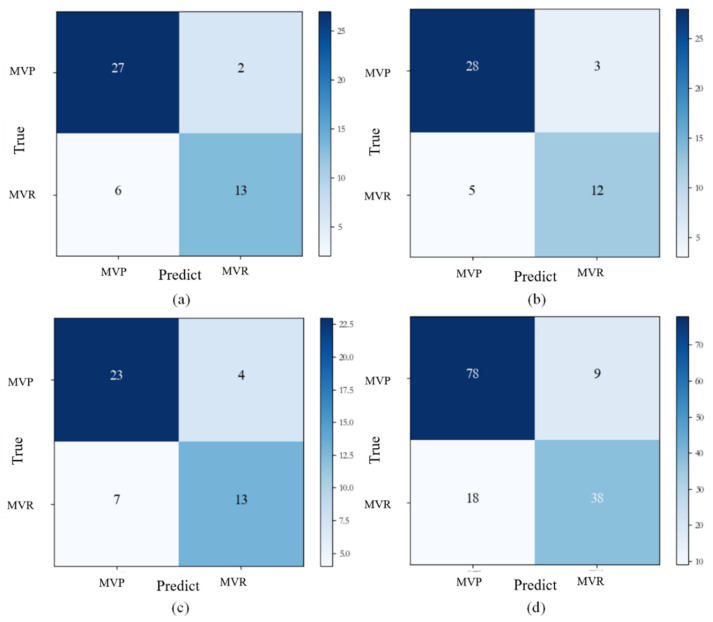
Confusion matrix for predicted outcomes. (**a**) Group 0; (**b**) Group 1; (**c**) Group 2; (**d**) total data. Abbreviations: MVR: mitral valve replacement, MVP: mitral valve repair.

The experimental results showed that the established model had an accuracy of 81.09% (0.833, 0.833, and 0.766 for Groups 0, 1, and 2, respectively) in predicting the surgical approach for patients. The confusion matrix diagram of the predicted results is shown in Figure 12.

### 3.6. Feature Importance

**Figure 13:**
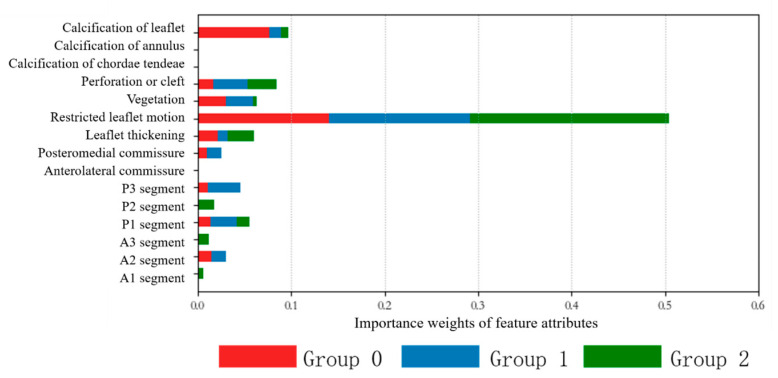
Importance weights of feature attributes. The top three characteristic attributes obtained for Group 0 were limitation of leaflet movement (42.04%), leaflet calcification (23.0%), and the presence of redundancy (8.94%). The top three characteristics obtained for Group 1 were restriction of leaflet movement (45.42%), presence of leaflet perforation or fissure (10.99%), and cumulative P3 sector of prolapse (10.54%). The top three characteristic attributes obtained for Group 2 were limitation of leaflet movement (50.42%), leaflet calcification (9.66%), and the presence of leaflet perforation or fissure (8.39%).

To determine the importance of feature attributes, the feature importance predicted by the model was extracted, as shown in Table 2. The results of the model algorithm showed that the three main factors affecting the choice of surgery were restricted leaflet motion, leaflet calcification, and perforation or cleft. The importance weights of the feature attributes on the influencing factors of the lesion, which were obtained using the superposition of the three results, are shown in Figure 13. The importance weights of the features were 50.4%, 9.66%, and 8.39% in Groups 0, 1, and 2, respectively.

## 4. Discussion

### 4.1. Proportion of Disease Components

In this group of patients, mitral valve prolapse was the most common etiology, which was consistent with reports of related epidemiological investigations on mitral valve regurgitation [3]. Most of the patients in this study were young or middle-aged, with fewer elderly patients, which may have affected whether the patients chose surgical treatment. Mitral valve prolapse was the most common etiology in this study and the P2 segment was the most frequently involved, which is related to the anatomical structure of the mitral valve apparatus and was consistent with relevant literature reports [19]. Infective endocarditis was the second most common cause in this study, mainly in young people, and inflammation mainly resulted in valve perforation and the formation of vegetation.

### 4.2. Correlation between the Complexity Score and Surgical Technique Score

Among the patients undergoing mitral valve repair, more complicated lesions needed the use of more complicated surgical techniques during the operation. The surgical technique score was correlated with the complexity score (r = 0.6317, *p* < 0.0001) (Supplementary Materials Figure S1). For example, the most common etiology in the simple group was atrial mitral regurgitation. In the vast majority of cases, only implantation of an annuloplasty ring can reduce the area of the annulus and increase the area of valve coaptation, thus reducing or even eliminating mitral regurgitation. In the intermediate group, for patients with mitral valve prolapse with involved multiple segments, in addition to implanting an annuloplasty ring, reconstructing the chordae tendineae and redundant leaflet resection was needed to provide enough support in systole and increase the area of valve coaptation. Rheumatic valvular disease was the most common cause in the complex group (70.5%). Other causes included mitral valve prolapse involving multiple segments (11.40%), Barlow’s syndrome (6.8%), and infective endocarditis (11.4%), of which the proportion of patients with each disease was almost the same. These diseases in the complex group not only involved a wide range of leaflets but were also accompanied by changes in valve morphology and chordae tendineae, which greatly increased the difficulty of surgery. In addition to conventional treatments, such as implanting an annular ring and reconstructing chordae tendineae, other complex operations, such as junction incision and leaflet resection, were also needed. It is also difficult to adjust the length of artificial chordae tendineae to achieve the best attachment. Therefore, the rate of mitral valve repair in the simple group was high (89.5%, 34/38), but significantly decreased in the intermediate (35.6%, 16/45) and complex (25.7%, 9/35) groups. Additionally, the intermediate and complex groups were more likely to convert to artificial valve replacement.

### 4.3. Advantages of Building a Prediction Model for Surgical Approach Based on the XGBoost Algorithm

Most previous studies have focused on the surgical influencing factors of a single type of mitral valve disease [17,23,24] in which mitral valve prolapse was common, while few studies have included an analysis of multiple common mitral valve lesions. Few studies have used a specific feature of echocardiography as a predictor for common mitral valve diseases or surgical planning for patients. Therefore, a predictive model for a variety of mitral valve diseases, based on multiple echocardiographic features, is urgently required to assist surgeons in surgical planning. In this study, the XGBoost algorithm was used to create a model to predict the appropriate surgical approach (mitral valve repair or replacement) according to the detailed echocardiographic features of mitral valve disease.

As shown in this study, the traditional complexity score was used to predict the surgical method with a rate of accuracy of 68.9%. Compared to the traditional complexity score, the XGBoost algorithm improved the accuracy by approximately 12%, with an accuracy of 81.09%. The reasons for this result may be as follows: first, the performance of XGBoost was robust [14,25]. XGBoost is widely used in many data science competitions; for example, Kaggle. The second reason is convenient transplantation [15,16]. XGBoost is supported on various distributed environments, such as Kubernetes, Hadoop, and SGE.

In addition to its high detection accuracy and convenient transplantation, another advantage of XGBoost is its analysis of the importance of influencing factors, which determined the surgical approach in our task. This information can provide an auxiliary reference for surgical methods, especially for difficult cases in the future. Depending on the etiology, the factors influencing the success rate of mitral valve repair were varied. Through the prediction model in this study, the key lesions were determined to be restricted leaflet motion, leaflet calcification, and perforation or cleft of the leaflet, indicating that when these types of lesions occur, the success rate of mitral valve repair may be lower. Additionally, cardiac surgeons need to choose the surgical approach carefully and seriously. The above three lesions are common in patients with rheumatic valvular disease and infective endocarditis, in which cardiac surgeons have great difficulty in repairing mitral valves [26,27,28].

Restricted valve motion and the calcification of leaflets are more common in patients with rheumatic heart disease; these conditions lead to thickening and calcification of leaflet tissue, poor valve elasticity, and fusion of commissures due to the repeated effects of rheumatism [29]. In addition to the fusion of commissures, it is necessary to release the fused chordae tendineae or reconstruct the chordae tendineae during the operation, which is difficult to perform and has a low success rate [10,26,27]. Due to the loss of the original shape of the leaflet, it is difficult to restore normal leaflet function to the valve (i.e., prelesion state) using surgical treatments, such as trimming the valve, removing the thickened leaflet tissue, and releasing the contracture chordae tendineae under the valve. According to a long-term follow-up study, the short-term outcomes for rheumatic valvular heart disease after mitral valve repair are good, but the long-term effect is not satisfactory. Even after receiving surgical treatment, patients with rheumatic heart disease still have inflammatory factors related to rheumatic fever in the body, so the valve structure is still affected by inflammatory factors and reactions that aggravate the lesions [12]. Mitral valve replacement in patients with significant valve calcification and abnormal tendinous chordae may be a better choice for long-term outcomes.

Perforation or cleft may occur in patients with congenital valve dysplasia or infective endocarditis. For patients with infective endocarditis, the field of vision should be fully exposed during surgery to display leaflet perforation, vegetation of various sizes, and extended infection [26]. Inflammation can damage valve tissue, causing valve destruction and a reduction in the healthy valve area, resulting in poor coaptation and massive regurgitation. For such patients, there is limited normal valve leaflet tissue available to repair the leaflet and re-establish a sufficient coaptation area during the operation, which requires an experienced surgeon to solve. In addition, for patients with infective endocarditis, the main purpose of surgical treatment is to completely remove the infected tissue in the heart. If the infected tissue is not completely removed during surgery, secondary infection may occur, leading to postoperative complications [26]. Therefore, not only are experienced surgeons required, but the extent of valve lesions needs to be reported in detail by preoperative echocardiography to assist with surgical planning.

The three high-risk factors identified in our model, including restricted valve movement, calcification of leaflets, and perforation or cleft of the leaflet, indicated that the quality of the leaflet itself is the most important factor for the success of the operation. Even if repair is performed, the quality of the leaflet will make it difficult to obtain an ideal coaptation, which will ultimately affect the outcome. This focus on the weight of high-risk factors is very valuable in surgical planning for individual patients. For example, in Case 4, the patient had rheumatic valvular disease with a complexity score of 8, thus belonging to the intermediate group. According to the prediction of the traditional complexity score, mitral valve repair was planned and performed. However, intraoperative transesophageal echocardiography after resuscitation showed moderate mitral valve regurgitation. Meanwhile, the XGBoost prediction model revealed that this patient had high-risk factors, including restricted valve movement and thickened and shortened chordae tendineae, indicating that the appropriate surgical choice was mitral valve replacement.

Therefore, the prediction model established using XGBoost to select the surgical method according to the characteristics of mitral valve disease can provide a scoring system through the patient’s preoperative ultrasound examination to assist the surgeon in determining whether the patient is suitable for mitral valve repair. For some high-risk patients, the prediction model could help avoid a second cardiac arrest or postoperative complications caused by an inappropriate surgical approach, thus benefiting every patient with mitral valve disease.

## 5. Conclusions

Our predictive model using the XGBoost algorithm based on echocardiographic features could assist surgeons in deciding whether a patient with mitral valve disease should take mitral valve repair or replacement. Further studies are required to validate our predictive model in other cardiac surgery centers and evaluate the practical applications of our model.

## 6. Limitation and Strengths

The limitations of this study include the following: (1) This is a single-center retrospective study. (2) We did not quantitatively evaluate the pathological characteristics of the mitral valve. In further studies, this evolution is necessary to improve and establish a scoring system that can help select patients with mitral valve disease. In the future, we will conduct a multi-center study to improve the accuracy and robustness of the predictive model.

The strengths of this study include the following: (1) The patients who received mitral valve surgery in the same period had a variety of diseases, such as mitral valve prolapse, rheumatic valve disease, infective endocarditis, and other mitral valve diseases. (2) Because this was a single-center study, the standard for surgical plan formulation was relatively uniform. (3) Among patients who underwent mitral valve repair, a certain number of postoperative adverse events, such as water tests indicating massive regurgitation, moderate or greater regurgitation during follow-up, and recurrence of prolapse in one patient at follow-up, contributed to a better predictive model.

## Figures and Tables

**Table 1:** Calculating the complexity score.

Complexity Variable	Weight
**Prolapse segments**	
P1 segment	1
P2 segment	1
P3 segment	1
A1 segment	2
A2 segment	2
A3 segment	2
Anterolateral commissure	2
Posteromedial commissure	2
**Ruptured chordae tendineae**	1
**Leaflet morphology**	
Normal	0
Thickening	1
Redundant	3
**Calcification**	
Leaflet	1
Annulus	3
Chordae tendineae	3
Fusion of commissure	2
**Perforation or Cleft**	
1	1
≥2	2
**Vegetation**	
1	1
≥2	2
**Leaflet Motion**	
Normal	0
Excessive	1
Restriction	2

Complexity score = sum of weights. Complexity strata (by complexity score): simple: 1–4; intermediate: 5–8; complex: ≥9.

**Table 2:** Echocardiographic features included in the XGBoost model.

Reference Variable	Class	Detailed Parameters
**Effective variables**		
Ruptured chordae tendineae	2	Yes; No
Leaflet morphology	3	Normal; Thickening; Redundant
Leaflet motion	2	Normal; Hypermobility; Restriction
Vegetation	3	0; 1; ≥2
Perforation or Cleft	3	0; 1; ≥2
Calcification	4	Annulus; Leaflet, Chordae tendineae; Fusion of commissure
**Uncertain variables**		
Prolapsed segments	8	P1 segment; P2 segment; P3 segment; A1 segment; A2 segment; A3 segment; Anterolateral commissure; Posteromedial commissure
**Ineffective variables**		
Sex	2	Man; Female
Age	-	-
Diagnosis	6	Mitral valve prolapses; Barlow’s syndrome; Rheumatic heart valve disease; Infective endocarditis; Atrial mitral regurgitation and other causes of mitral valve regurgitation or stenosis

**Table 3:** The basic clinical and echocardiographic features of patients in three different groups.

Variable	Simple (Score: 1–4) (*n* = 38)	Intermediate (Score: 5–8) *n* = 61	Complex (Score: ≥9) *n* = 44
**Sex**			
Male	23 (60.5)	41 (67.2)	24 (54.5)
Female	15 (39.5)	20 (32.8)	20 (45.5)
**Surgical Approach**			
Mitral Valve Repair	33 (86.8)	45 (73.8)	9 (20.5)
Mitral Valve Replacement	5 (13.2)	16 (26.2)	36 (79.5)
**Diagnosis**			
Mitral Valve Prolapse	12 (31.6)	38 (62.3)	5 (11.4)
Infective Endocarditis	6 (15.8)	13 (21.3)	5 (11.4)
Rheumatic Heart Disease	0	8 (13.1)	31 (70.5)
Atrial mitral regurgitation	16 (42.1)	0	0
Barlow’s Syndrome	0	1 (1.6)	3 (6.8)
Others	4 (10.5)	1 (1.6)	0
**Prolapsed segments**			
A1 segment	2 (5.3)	10 (16.4)	5 (11.4)
A2 segment	0	20 (32.8)	8 (18.2)
A3 segment	0	16 (26.2)	9 (20.5)
P1 segment	3 (7.9)	10 (16.4)	3 (6.8)
P2 segment	10 (26.3)	26 (42.6)	5 (11.4)
P3 segment	4 (10.5)	16 (26.2)	7 (15.9)
Anterolateral commissure	0	0	0
Posteromedial commissure	0	10 (16.4)	5 (11.4)
**Ruptured chordae tendineae**			
No	36 (94.7)	38 (62.2)	35 (79.6)
Yes	2 (5.3)	23 (37.8)	9 (20.4)
**Leaflet morpholopy**			
Normal	33 (86.8)	27 (44.3)	0
Redundant	0	1 (1.6)	3 (4.5)
Thickening	5 (13.2)	33 (54.1)	42 (95.5)
**Leaflet motion**			
Normal	25 (65.8)	0	0
Excessive	13 (34.2)	52 (85.2)	10 (22.7)
Restriction	0	9 (14.8)	34 (77.3)
**Vegetation**			
0	34 (89.5)	47 (77.0)	41 (93.2)
1	4 (10.5)	10 (16.4)	2 (4.5)
≥2	0	4 (6.6)	1 (2.3)
**Perforation or Cleft**			
0	37 (97.4)	53 (86.9)	41 (93.2)
1	1 (2.6)	7 (11.5)	1 (2.3)
≥2	0	1 (1.6)	2 (4.5)
**Calcification**			
Annulus	0	0	0
Leaflet	0	1 (1.6)	31 (70.5)
Chordae tendineae	0	8 (13.1)	31 (70.5)
Fusion of Commissure	0	8 (13.1)	31 (70.5)

Values are *n* (%), unless otherwise indicated.

**Table 4:** Treatment and outcomes of patients in each group.

Endpoint	Simple (*n* = 38)	Intermediate (*n* = 61)	Complex (*n* = 44)
Mitral valve repair	33	45	9
Mitral valve replacement	5	16	35
Unsuccessful repair	4	6	5
Diverted to mitral valve replacement	3	4	4
More than mild regurgitation during follow-up	1	2	1

**Table 5:** ROC curve parameters based on complexity scores.

AUC	Cut-Off Value	1-Specificity	Sensitivity	True Positive	True Negative	False Negative
0.75	8.5	0.33	0.73	0.73	0.67	0.33

## Data Availability

The raw data required to reproduce these findings cannot be shared at this time as the data also forms part of an ongoing study.

## Supplementary Materials

The following supporting information can be downloaded at: https://www.mdpi.com/article/10.3390/jcm12031193/s1, Figure S1: The correlation between complexity score and surgical technique score.
