# Supplementary material for: Prediction of Surgical Approach in Mitral Valve Disease by XGBoost Algorithm Based on Echocardiographic Features
Source: J Clin Med. 2023 Feb 2;12(3):1193. doi: 10.3390/jcm12031193 (PMC9917697; doi:10.3390/jcm12031193)
Supplement: Supplementary file 1 [file jcm-12-01193-s001.zip › jcm-2109576-supplementary.pdf]

Supplementary Materials

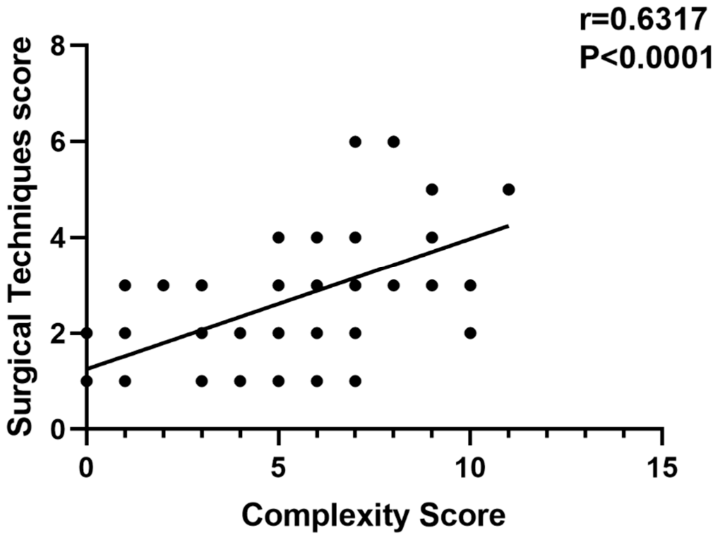

Figure S1. The correlation between complexity score and surgical technique score.
